# Editorial: Filling the research gaps of malaria pathobiology

**DOI:** 10.3389/fmicb.2026.1880918

**Published:** 2026-05-28

**Authors:** Wenn-Chyau Lee, Tadesse Hailu, Letusa Albrecht

**Affiliations:** 1 Department of Parasitology, Faculty of Medicine, Universiti Malaya, Kuala Lumpur, Malaysia; 2 A*STAR Infectious Diseases Labs (A*IDL), Agency for Science, Technology and Research (A*STAR), Singapore, Singapore; 3 Department of Medical Parasitology and Entomology, College of Medicine and Health Sciences, Bahir Dar University, Bahir Dar, Ethiopia; 4 Laboratório de Pesquisa em Apicomplexa—Instituto Carlos Chagas, Fundação Oswaldo Cruz, Curitiba, Brazil

**Keywords:** epidemiology, malaria, molecular parasitology, pathobiology, transmission biology

## Introduction

Malaria remains a formidable global health crisis, claiming hundreds of thousands of lives each year despite intensive intervention (World Health Organization, 2024). The landscape of this tropical infection is further complicated by the rise of potentially fatal zoonotic malaria transmission in Southeast Asia, which presents distinct transmission dynamics and pathobiology (Cox-Singh et al., 2010; Lau et al., 2016; Lee et al., 2022a,b; Masse et al., 2025). The parasite's sophisticated lifecycle, high adaptability to stress, and its uncanny ability to remodel host environments present a moving target for eradication efforts (Fowkes et al., 2024; Balmer et al., 2025). While our foundational knowledge is vast, significant “pathobiological gaps” persist—hidden mechanisms of survival, transmission, and host evasion that continue to undermine current strategies. This Research Topic, “*Filling the research gaps of malaria pathobiology*,” curates six pivotal contributions that bridge these divides, offering fresh insights into the molecular vulnerabilities and ecological realities of *Plasmodium*.

In the realm of medical parasitology, identifying molecular vulnerabilities—specific genetic or metabolic weaknesses of the parasites that allow for the selective killing of parasites—remains a cornerstone of novel treatment discovery. Tashie et al. discuss the potential of targeting *Plasmodium falciparum* Equilibrative Nucleoside Transporters (*Pf* ENTs) to develop purine-analog-based antimalarials. Complementing this, Wakai et al. review the biology of *Plasmodium* telomere maintenance as an “Achilles' heel” for developing parasite-specific therapeutics. However, bridging the gap between potential and reality requires rigorous validation, as exemplified by the study by Chakraborty et al. on *P. falciparum* MKT1 domain proteins. The study provides a critical reality check, demonstrating that not every conserved parasite protein is a feasible target against the *Plasmodium* erythrocytic stages. This finding underscores the importance of functional genomics in narrowing our focus toward elements truly essential for parasite survival.

The cellular biology of parasite survival also hinges on complex interactions between parasite- and host-derived factors. Chin et al. explore how the human periostin (OSF-2) protein influences the phagocytic clearance of erythrocytes infected with *P. falciparum* and *P. knowlesi*, offering a rare look at the host-mediated defense mechanisms. Parallel to survival is the “escape hatch” of transmission; Subramaniam et al. review the induction of gametocytogenesis, detailing how environmental stress and genome editing can be applied to manipulate the production of parasite sexual stages. Together, these writings provide a multi-faceted view of how we might disrupt the parasite biology and the infection pathobiology.

Finally, laboratory-based insights must be grounded in epidemiological reality. Daud et al. demonstrate varied serological responses to different *P. falciparum*-derived proteins in a near-elimination setting. Their work provides crucial information regarding residual malaria transmission, highlighting the need for location-targeted interventions that move beyond the “one-size-fits-all” elimination strategies.

This Research Topic brings together a concise yet impactful collection of works that address critical voids in our understanding of *Plasmodium* biology. From the molecular—exploring the essentiality of MKT1 domain proteins and the therapeutic potential of purine antimetabolites—to the epidemiological, where serological data reveals the hidden persistence of malaria, these works underscore a singular truth: filling research gaps requires a multi-disciplinary lens. By bridging the gap between basic molecular machinery and complex host-parasite dynamics, this Research Topic provides a roadmap for the next generation of antimalarial strategies and malaria management.
